# The Spatiotemporal Development of Intercalated Disk in Three-Dimensional Engineered Heart Tissues Based on Collagen/Matrigel Matrix

**DOI:** 10.1371/journal.pone.0081420

**Published:** 2013-11-15

**Authors:** Jin Zhou, Yao Shu, Shuang-Hong Lü, Jun-Jie Li, Hong-Yu Sun, Rong-Yu Tang, Cui-Mi Duan, Yan Wang, Qiu-Xia Lin, Yong-Chao Mou, Xia Li, Chang-Yong Wang

**Affiliations:** 1 Department of Advanced Interdisciplinary Studies, Institute of Basic Medical Sciences and Tissue Engineering Research Center, Academy of Military Medical Sciences, Beijing, China; 2 Laboratory of Oncology, Affiliated Hospital of Academy of Military Medical Sciences, Beijing, China; National University of Ireland, Galway, Ireland

## Abstract

Intercalated disk (ID), which electromechanically couples cardiomyocytes into a functional syncitium, is closely related to normal morphology and function of engineered heart tissues (EHTs), but the development mode of ID in the three-dimensional (3D) EHTs is still unclear. In this study, we focused on the spatiotemporal development of the ID in the EHTs constructed by mixing neonatal rat cardiomyocytes with collagen/Matrigel, and investigated the effect of 3D microenvironment provided by collagen/Matrigel matrix on the formation of ID. By histological and immmunofluorescent staining, the spatiotemporal distribution of ID-related junctions was detected. Furthermore, the ultra-structures of the ID in different developmental stages were observed under transmission electron microscope. In addition, the expression of the related proteins was quantitatively analyzed. The results indicate that accompanying the re-organization of cardiomyocytes in collagen/Matrigel matrix, the proteins of adherens junctions, desmosomes and gap junctions redistributed from diffused distribution to intercellular regions to form an integrated ID. The adherens junction and desmosome which are related with mechanical connection appeared earlier than gap junction which is essential for electrochemical coupling. These findings suggest that the 3D microenvironment based on collagen/Matrigel matrix could support the ordered assembly of the ID in EHTs and have implications for comprehending the ordered and coordinated development of ID during the functional organization of EHTs.

## Introduction

The last decade has witnessed great improvement in cardiac tissue engineering, and a variety of biomaterials, including naturally polymers, synthetic polymers and decellularized extracellular matrix (ECM), have been developed to construct cardiac tissues with rhythmic contraction *in vitro* [1]–[4]. Notably, highly organized sarcomeres and specialized intercellular junctions, i.e., intercalated disk (ID) can be observed in engineered heart tissues (EHTs), suggesting that some ultra-structural features of EHTs resemble that of native myocardium [5], [6].

ID, composed of adherens junctions, gap junctions and desmosomes, is a complex and highly orchestrated structure. Via ID, cardiomyocytes are electromechanically coupled into a functional syncitium, and hence it is essential for normal morphology and function of myocardium [7]. Therefore, the formation of specialized ID may be closely associated with the quality and function of EHTs. And current research has demonstrated that adherens junctions and/or gap junctions can be detected by the expression of their hallmark proteins in the EHTs [3], [8].

Furthermore, the studies *in vivo* and *in vitro* have proven that there is a close relationship among adherens junctions, gap junctions and desmosomes. The mutation of even one single protein would influence structural integration of the disk and cardiac function [7], [9], [10]. Meanwhile, with hallmark proteins, the development mode of ID in primary cultured cardiomyocytes is investigated. The three junctions are organized in a precise spatial and sequential manner and the assembly of adherens junctions and desmosomes precedes the formation of gap junctions [11], [12]. However, until now, the intact and ordered formation of the ID in EHTs has not been thoroughly investigated.

Since cardiomyocytes in EHTs lived in a 3D microenvironment provided by biomaterials, the formation mode of the ID during the remodeling of cardiomyocytes may be affected by the used biomaterials. Collagen and Matrigel are common natural materials used in tissue engineering. Collagen is a main component of myocardial ECM and one of optimal biomaterials for the construction of EHTs [13]. Matrigel is an available matrix purified from mouse sarcoma cells which contains a number of basement membrane proteins and some growth factors. It is often used to create a bioactive environment with multiple growth factors [13]. Hence, the composite of collagen and Matrigel is a favorite scaffold in cardiac tissue engineering, which has been applied in a series of EHTs [5], [6], [14]. In our laboratory, EHTs have been successfully constructed the by mixing neonatal rat cardiomyocytes with collagen/Matrigel [6], [15]. However, the formation pattern of the ID in collagen/Matrigel matrix has not been explored. In order to better understand the formation process of EHTs, the time course of expression and distribution of ID-associated proteins in collagen/Matrigel matrix were investigated in this study, moreover, the ultra-structural sequential patterns of ID formation was also studied.

## Materials and Methods

All animal experiments were carried out under the guidelines of the Institutional Animal Care and Use Committee of the Chinese Academy of Military Medical Science (Beijing, China). The protocol was approved by the Committee on the Ethics of Animal Experiments of the Chinese Academy of Military Medical Science. All surgery was performed via 4–5% isoflurane inhalation, and all efforts were made to minimize suffering.

### Cell Culture

Neonatal rat cardiomyocytes were obtained from 0- to 3-day-old SD rats as previously described [15]. The collected cell pellet was resuspended in media and pre-plated for 1 h in DMEM culture medium to enrich for cardiomyocytes by allowing attachment of fibroblasts. Unattached cells (6×10^5^/ml) were cultivated on coverslips in 24-well plates, and the culture medium was changed every other day.

### Construction of the EHTs

The EHTs were constructed as previously described [15]. Briefly, the concentrated 2×DMEM culture medium (GIBCO), the liquid type I collagen from rat tails (2.4 mg/ml in 0.1 wt% acetic acid) and the basement membrane matrix (Matrigel; Becton Dickinson Biosciences) were mixed in 5∶4∶1 (v/v) at 0°C. The pH was adjusted to XX using 0.1 M NaOH. Then, freshly isolated cardiomyocytes from neonatal Sprague-Dawley rats (1–3 days old) were added into the mixture (8×10^6^ cells/ml) and thoroughly mixed. At last, the mixture was pipetted into the self-made moulds (1 mL/well) for pre-culturing in a 37°C/5%CO_2_ humidified incubator. After 0.5–1 h, 1 ml the DMEM culture medium was added and changed every day. The self-made moulds were made based on 12-well plates. Molten agar was poured into the plate and solidified quickly, and then four glass columns were inserted into ager and positioned properly to supply static stretch.

### Histology, immmunofluorescent staining and quantification [12]

The EHTs were fixed in 4% paraformaldehyde for 12–24 h, embedded in paraffin, sectioned at 2 µm, and stained with hematoxylin/eosin (H & E) for general evaluation.

For immmunofluorescent staining, neonatal (0–2 days old) and adult (250 g±20 g) rat hearts, the EHTs and neonatal rat cardiomyocytes in primary culture were processed and the sections/coverslips were incubated with primary antibodies in PBS containing 0.3 wt% Triton X-100 and 1 wt% bovine serum overnight at 4°C. Subsequently, the sections were incubated for 1 h at room temperature with different FITC-conjugated or Cy3-labeled secondary antibodies (goat anti-mouse IgG and goat anti-rabbit IgG, Boster china). The samples were assessed under a confocal laser-scanning microscope (CLSM, Carl Zeiss). The following primary antibodies were used: mouse anti-cardiac troponin T (abcam), anti-N-cadherin (abcam) and anti-plakoglobin (Sigma); rabbit anti-plakophilin2 (abcam) and anti-connexin43 (abcam).

The samples of each timepoint were exposed to PBS instead of primary antibodies, incubated sequentially using the same protocol, served as a negative control and run in parallel during each quantitative experiment. All processing procedures were performed under identical conditions, and the confocal settings were standardized for all parallel groups. The fluorescence intensity was quantified using Volocity Demo 6.1.1 software. Each experiment was repeated at least three times.

### Transmission electron microscopy (TEM) [12]

The EHTs were fixed in 2 wt% paraformaldehyde and 2.5 wt% gluteraldehyde overnight at 4°C, postfixed in 1 wt% osmium tetroxide, dehydrated in graded ethanol, and embedded in Epon 812. Ultra-thin sections were cut and stained with lead citrate and uranyl acetate and examined with a Philip Technai 10 transmission electron microscope.

### Western blotting

The EHTs were lysed in Laemmli Sample Buffer (Bio-Rad), and further homogenized with a rotor stator homogenizer. The extracted proteins were measured by the BCA™ Protein Assay Kit (Thermo Scientific). The equal quantities of proteins were loaded on a 10 wt% SDS-polyacrylamide gel and separated electrophoretically, and then were transferred to a PVDF membrane (Roche). Membrane was blocked with 5% defatted milk for 1 h at room temperature and then incubated with primary antibody overnight at 4°C (mouse anti-GAPDH, anti-N-cadherin and anti-plakoglobin; rabbit anti-connexin43 and anti-plakophilin2) and with appropriate horseradish peroxidase-coupled secondary antibodies for 1 h at room temperature (goat anti-mouse IgG and goat anti-rabbit IgG, Cell Signaling Technology). The signals of protein bands were detected by enhanced chemiluminescence reagent (Applygen). Band intensity was normalized to GAPDH's.

### Statistical analysis

All data are expressed as mean ± SD from at least 3 independent experiments. The data from the quantitative expression of the related proteins at different time point were compared and inter-group differences were analyzed by one-way ANOVA with Tukey's post-hoc test. Statistical analyses were performed with SAS 9.1. A value of *p*<0.05 was considered statistically significant.

## Results

### Remodeling of cardiomyocytes in the EHTs

**Figure 1:**
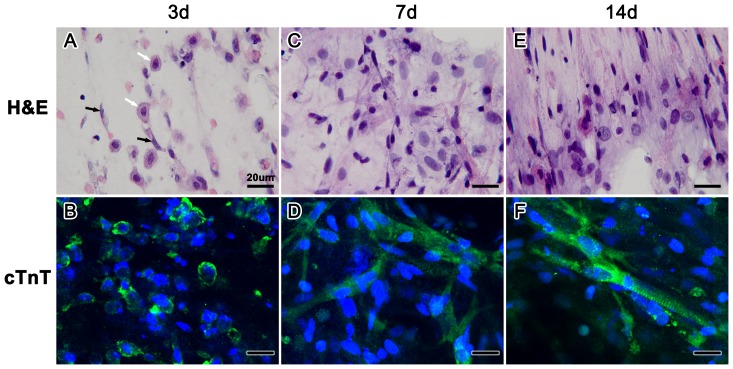
H & E and immunofluorescent staining of the EHTs cultured for 3, 7 and 14 days. (**A–B**) Oval-shaped cardiomyocytes (white arrow) and fibroblasts with the spreading pseudopods (black arrow). (**C–D**) A progressive increase in cell size and cTnT positive cardiomyocytes with the extensive pseudopods. (**E–F**) The aligned rod-shaped cells and well-arranged cTnT positive cardiomyocytes with clear sarcomeres.

The neonatal rat cardiomyocytes lost their classic morphology in collagen/Matrigel matrix at first, and most cells shrank to spheroids. After 3 days of culture, the cardiomyocytes began to remodel and spread inside the matrix. Most cardiac troponin T (cTnT) positive cardiomyocytes in collagen/Matrigel matrix became oval cells; meanwhile a few cells (e.g. fibroblasts) showed the spreading pseudopods (Fig. 1A–B). Over the course of 7 days, the cells in collagen/Matrigel matrix gradually extended their pseudopods to form new cell-matrix and cell-cell contacts (Fig. 1C–D), assembled new myofibrils to keep spontaneous beating, and finally regained the typical rod-like morphology. As shown in Fig. 1d, cTnT positive cardiomyocytes exhibited a rod-shape with extensive processes at day 7. After 14 days *in vitro*, cardiomyocytes further developed in collagen/Matrigel matrix and the degree of cell alignment increased dramatically (Fig. 1E–F). At day 14, highly expressed cTnT and clear sarcomeres could be detected in cardiomyocytes in the collagen/Matrigel matrix (Fig. 1F).

### Spatiotemporal distribution of ID-related proteins in the EHTs

To identify and characterize ID-related structures, the marker proteins (N-cadherin for adherens junctions, plakoglobin (PG) and plakophilin2 (PKP2) for desmosomes, and connexin43 (Cx43) for gap junctions) were detected in the EHTs cultured for 3, 7 and 14 days, respectively. And then the expression of ID-related proteins in the EHTs at different time point was compared with that in primary cultured neonatal rat cardiomyocytes, native myocardium of neonatal and adult rats, respectively. In addition, the fluorescence intensity was analyzed to quantify the expression of ID-related proteins in both 3D and 2D culture.

**Figure 2:**
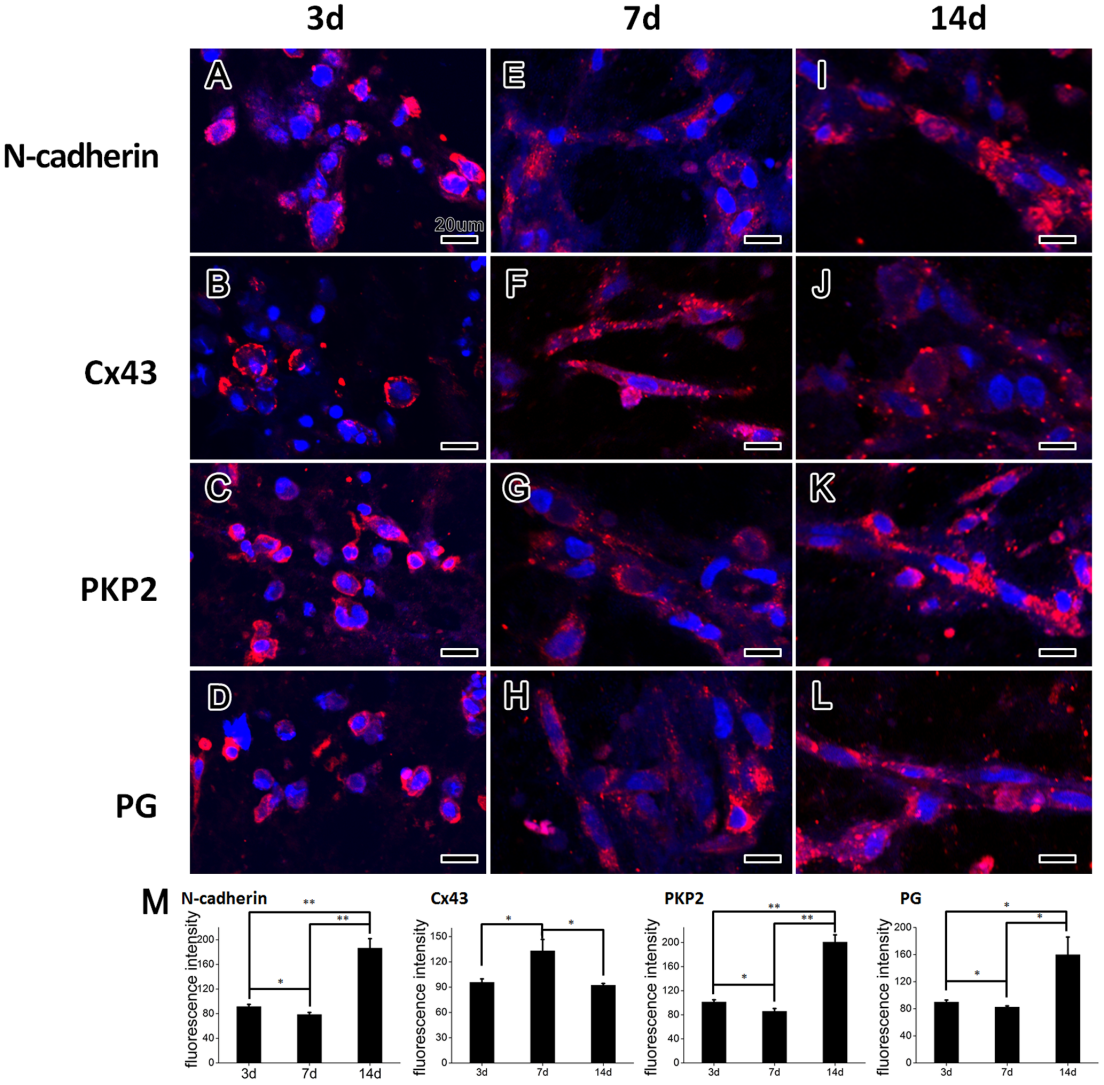
Immunofluorescent staining and quantification of ID-related proteins of the EHTs cultured for 3, 7 and 14 days. (**A–D**) At day 3, the cell membranes were positively stained with N-cadherin, Cx43, PKP2 and PG. (**E–H**) At day 7, N-cadherin (**E**), PKP2 (**G**) and PG (**H**) staining were positive and mainly localized to the contact sites of adjacent cells (arrow). Whereas the Cx43 positive staining was distributed along the whole plasma membrane in a punctuate pattern (**F**). (**I–L**) At day 14, ID-related proteins concentrated at the polar ends (arrow) of cardiomyocytes. (**M**) The FI of N-Cadherin, PKP2 and PG decreased from day 3 to 7 but increased at day 14. Cx43 was expressed the opposite. **p<0.01; *p<0.05.

At day 3, cardiomyocytes in collagen/Matrigel matrix were oval shaped. Most of N-cadherin (Fig. 2A), Cx43 (Fig. 2B), PKP2 and PG (Fig. 2C–D) were distributed in the entire plasma membrane.

During 7 days of culture, along with the cell growth in collagen/Matrigel matrix, cardiomyocytes extensively contacted with each other. Therefore, ID-related proteins were highly expressed and organized in the collagen/Matrigel matrix. N-cadherin gradually localized towards the intercellular regions (Fig. 2E). Similarly, PKP2 and PG were also detected in the interface of neighboring cells (Fig. 2G, H). By contrast, Cx43 was mainly located along the the whole cell membrane in a punctuate pattern, but rarely localized in the intercellular regions (Fig. 2F). This indicates that N-cadherin, PKP2 and PG may appear earlier than Cx43 during the development of the ID.

After 14 days of culture, with well-organized alignment of cardiomyocytes in the collagen/Matrigel matrix, the hallmark proteins of adherens junction and desmosome, including N-cadherin, PKP2 and PG, accumulated at the polar ends of cells along a new longitudinal axis (Fig. 2I, K–L). Meanwhile, Cx43 protein for gap junction obviously shifted its position from cell membranes to cell-cell contact sites (Fig. 2J).

By comparing the fluorescence intensity (FI), we found that the mean value of FI of N-cadherin, PKP2 and PG slightly decreased from day 3 to 7, and then significantly increased at day 14 (Fig. 2M). By contrast, the FI level of Cx43 significantly increased from day 3 to 7, and decreased at day 14.

**Figure 3:**
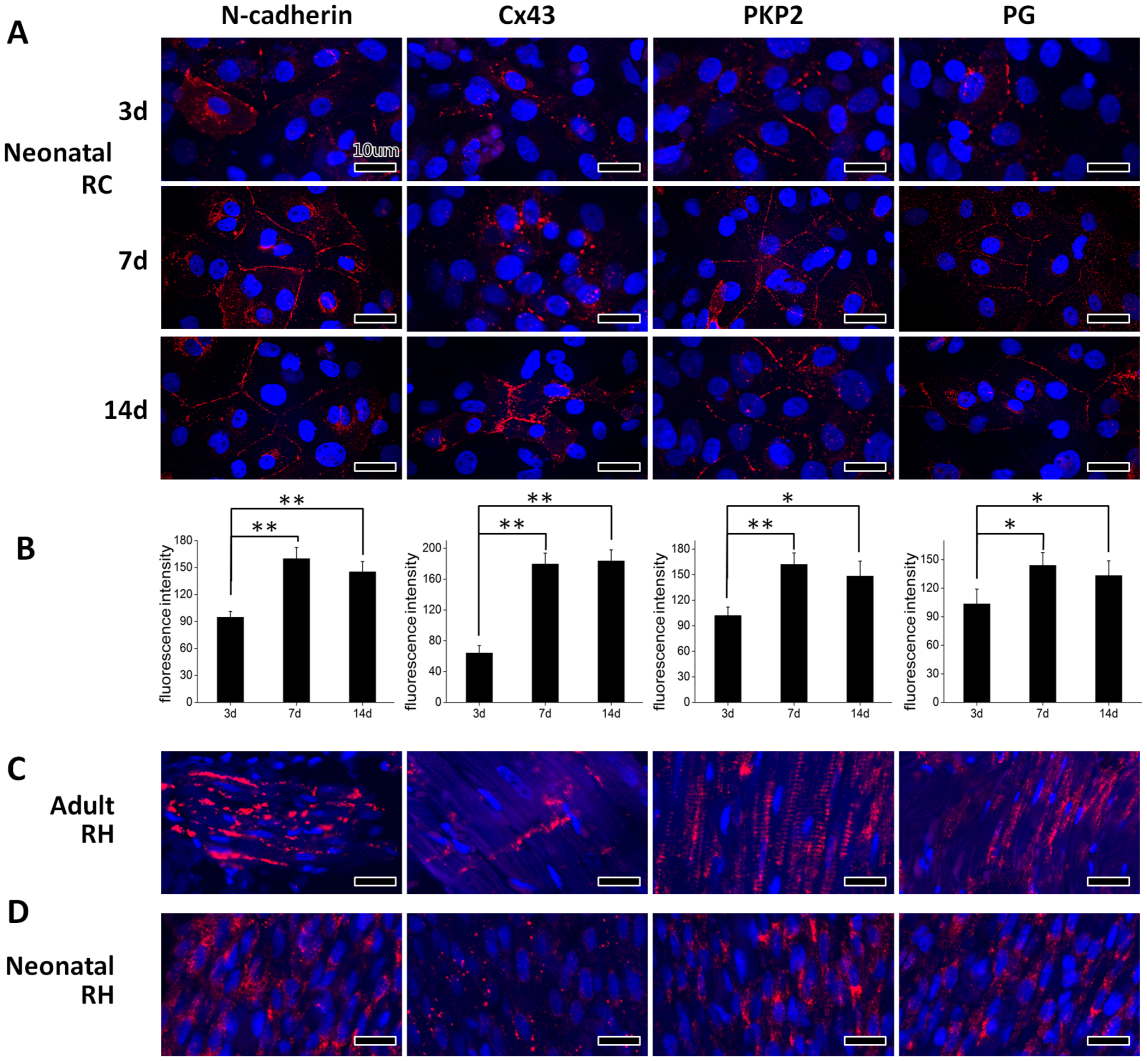
Immunofluorescent staining and quantification of ID-related proteins in primary cultured neonatal rat cardiomyocytes at different time point, adult and neonatal rat heart. (**A**) ID-related proteins were progressively changed their localization from the cells periphery to cell-cell contact sites. (**B**) ID-related proteins significantly increased at day 7 but slightly decreased (N-Cadherin, PKP2 and PG) or kept unchanged (Cx43) at day 14. **p<0.01; *p<0.05. (**C–D**) ID-related proteins accumulated in the contact sites of adjoining cells in neonatal and adult rat hearts. RC: rat cardiomyocytes; RH: rat heart.

Fig. 3A showed the distribution of ID-related proteins in primary culture of neonatal rat cardiomyocytes. At day 3, cardiomyocytes extended their pseudopods, and ID-related proteins scattered along the cell membrane. After 7 days of culture, cell-cell contacts were extensively formed, and ID-related proteins gathered in intercellular region in a linear distribution, except that Cx43 was still dispersed in a punctate distribution. At day 14, the intercellular contacts formed more localized structures, and all ID-related proteins were recruited to a more localized region. The results suggested that the spatiotemporal distribution of ID in 2D culture was similar with that in the EHTs (Fig. 2). In addition, the expression of ID-related proteins dramatically increased from day 3 to day 7, but slightly decreased or kept unchanged at day 14 in 2D culture system (Fig. 3B), which is different from that in the EHTs (Fig. 2M).

As shown in Fig. 3C, cardiomyocytes in cardiac tissues of adult rat exhibit long rod-like in a linear arrangement and ID-related proteins were confined to longitudinal or transversal intercellular regions. By contrast, ellipse-shaped cardiomyocytes in cardiac tissues of neonatal rats were closely arranged (Fig. 3D), and the ID-related proteins were extensively located in intercellular regions. Some characteristics of the cardiomyocytes and ID in day-14 EHT, e.g. well-extended and aligned–arranged cells, and intercellular distributions of ID proteins, were similar to those found in rat heart tissues.

### Spatiotemporal development of ultra-structures of the ID in the EHTs

**Figure 4:**
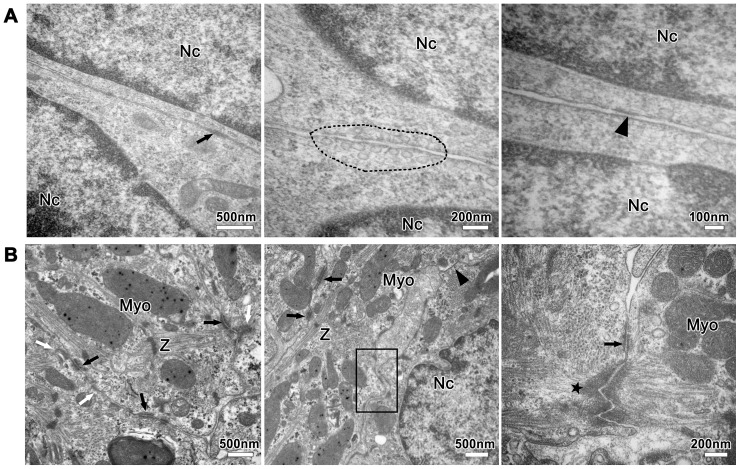
Ultra-structures of the nascent ID in the EHTs cultured for 3 and 7 days. (**A**) Closely apposed cell membranes at day 3 with scarce plaque-like structure (black arrow), subplasmalemmal vesicles (dotted-line area) and fibril-like structure (arrowhead). (**B**) Adherens junctions (white arrow) and desmosomes (black arrow) dispersed among the ID at day 7. New intercellular junctions were forming (black-line area) and a single gap junction profile appeared (arrowhead). Note an adhesion region with a dense plaque of adherens junction (star) anchoring actin filaments and a desmosome (black arrow). Myo: myofibril; Z: Z-line; Nc: nucleus.

On basis of the distribution pattern detected by immunohistochemical staining, we further observed the ultra-structures of the ID at different developmental stages under TEM. After 3 days of culture, the intercellular regions in collagen/Matrigel matrix displayed as a straight line, and no cytoskeleton filaments were observed (Fig. 4A). Under TEM, the early ID was characterized by parallel-arranged cell membranes and scarce plaque-like structures.

The ultra-structures of cardiomyocytes in the EHTs cultured for 7 days showed that the electron-dense plaques remarkably increased, and symmetrically gathered along the opposite plasma membranes of adjacent cells (Fig. 4B). Myofibrils were assembled to form immature sarcomeres with Z-lines and multiple mechanical junctions (arrows in Fig. 4B). But only a few gap junctions (arrowhead in Fig. 4B) were observed at cell-cell contact sites. Meanwhile new intercellular junctions were still forming, and the electron-dense plaques inclined to accumulate at subplasmalemmal sites between cells (black-line area in Fig. 4B). The developing ID with the fascia adherens-like dense plaque anchoring actin filaments (star in Fig. 4B) and the desmosome was visible. These features provided evidence for the later assembly of gap junction, consistent with the distribution mode of ID-related proteins at day 7 (Fig. 2).

**Figure 5:**
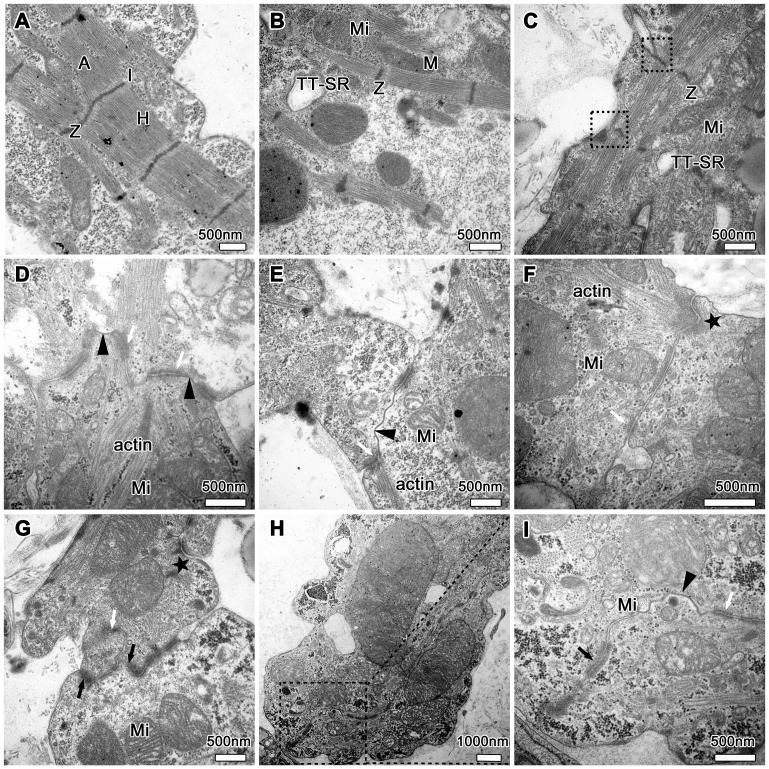
Ultra-structures of cardiomyocytes and well-developed ID in the EHTs cultured for 14 days. (**A–C**) Typical cross-striated sarcomeres with A-, I-, H-, M-, Z-bands and interspersed mitochondria (Mi). T tubule-SR (TT-SR) system and costamere structure (dotted-line) at the level of Z-band. (**D–I**) Adherens junctions (white arrow), desmosomes (black arrow) and short and large ribbon-like gap junctions (arrowhead) alternated with each other. Note numerous actin filaments (**D–F**) ended at the adherens junction, especially bundles of actin filaments inserting into dense plaques (star). Local magnification (**I**) of dotted-line area (**H**) indicated a high level of confluent and well-developed ID.

At 14 days after culturing in collagen/Matrigel matrix, rich mitochondria and glycogen granulae were positioned between myofibrils of cardiomyocytes (Fig. 5). Moreover, the important structural characteristic of cardiomyocyte terminal differentiation and cross-striated sarcomeres could also be observed via TEM (Fig. 5A–B). In detail, I, A, Z, M (immatured) and H (matured) bands were clearly distinguishable in the sarcomeres, indicating that cardiomyocytes in the EHTs were highly differentiated. T tubule-sarcoplasmic reticulum (SR) junctions in the form of dyads (Fig. 5B–C) and costamere structure (Fig. 5C) can also be observed, and the latter, as cell-matrix junction [16], was located at the level of Z band to connect the sarcomere and cell membrane.

As cardiomyocytes gradually developed in collagen/Matrigel matrix, the ID formed more ultra-structures at day 14. Cardiomyocytes in collagen/Matrigel matrix showed well-developed ID with complex of the three junctions (Fig. 5D–I). Furthermore, numerous and polymorphic gap junctions were assembled in the ID (Fig. 5D–E, H–I). Large ribbon-like gap junctions, resembling the morphology of gap junctions of adult rat cardiomyocytes *in situ* [17], could be observed (Fig. 5E, I), which indicated the maturation of cardiomyocytes to a certain extent.

### Quantitative Expression of ID-related proteins in the EHTs

**Figure 6:**
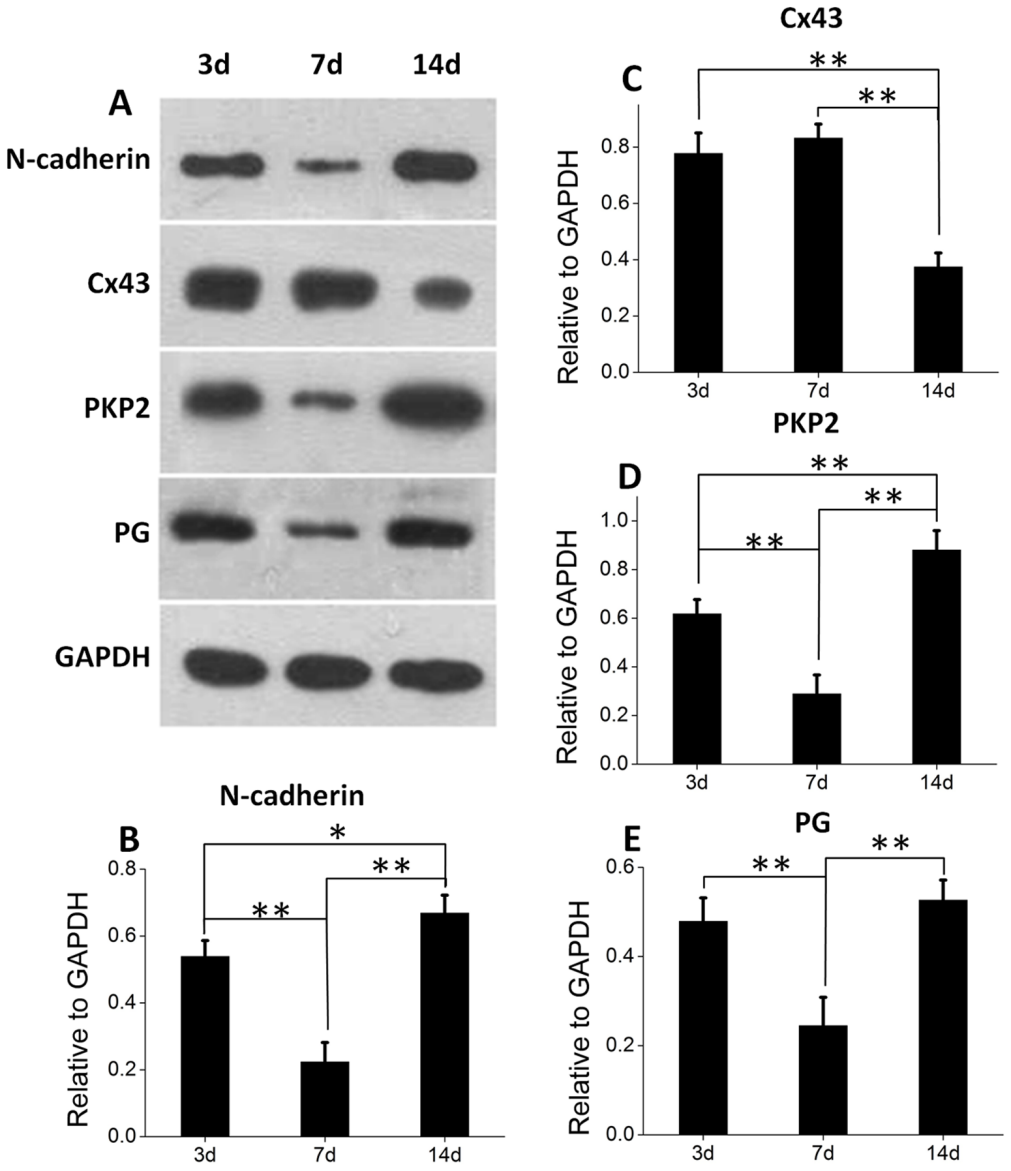
The quantitative expression of N-cadherin and Cx43 cultured for 3, 7 and 14 days. (**A, B, D, E**) N-Cadherin, PKP2 and PG significantly decreased at day 7 but significantly increased at day 14. (**A, C**) Cx43 increased a little at day 7 and then dramatically decreased at day 14. **p<0.01; *p<0.05.

The expression of N-cadherin and Cx43 which are closely related to mechanical and electrochemical connections was quantitative analyzed. As shown in Fig. 6, N-cadherin, PKP2 and PG were highly expressed at day 3, but significantly decreased at day 7, and then increased at day 14. In comparison, the expression level of Cx43 was high at day 3, which was similar with the other proteins at this time point, but increased at day 7 and dramatically decreased at day 14. The result was consistent with the data of quantitative immunofluorescence (Fig. 2M).

## Discussion

In this study, we investigated in detail the spatiotemporal development of the ID in the 3D microenvironment provided by collagen/Matrigel matrix. The results showed that along with the remodeling of cardiomyocytes in collagen/Matrigel matrix, ID-associated junctions(adherens junctions, desmosomes and gap junctions) gradually developed. The related proteins were successively distributed from the entire cell membranes to cell-cell interfaces, forming the integrated ID structure. And during this process, the assembly of N-cadherin for mechanical junctions preceded the formation of Cx43 for electrochemical junctions. This developing mode is related to the reconstruction of structure and contractile function of myocardium, because the earlier accumulation of N-cadherin reinforces cell-cell contacts and supplies enough close membrane space to facilitate the subsequent assembly of Cx43, and thus promote the formation of electrochemical coupling [12]. In contrast, redistribution of gap junctions from the ID region to the lateral sarcolemma, termed the lateralization of gap junctions, is a characteristic of a series of cardiac diseases [11], [18]. This pathological change is just right opposite with the distribution pattern in collagen/Matrigel matrix, which provides an additional evidence to demonstrate that the present results display a physiological distribution mode.

Basically, the spatiotemporal development in collagen/Matrigel matrix is consistent with that in the primary culture of neonatal (the control in this study and [19]) and adult [11], [12] rat cardiomyocytes, but there are still some difference. For example, the expression of N-cadherin, PKP2 and PG in 3D constructs significantly increased from day 7 to 14, but at this time point, the expression of these proteins in 2D culture was decreased or unchanged. In addition, at day 14, cardiomyocytes in the 3D constructs showed some terminal differentiation features, especially new-formed T tubule-SR dyads, which are absent in neonatal rat hearts [20] and in primary culture of adult rat cardiomyocytes [21]. The above results suggest that the 3D constructs based on collagen/Matrigel matrix are helpful for the expression of ID-related proteins and the development of neonatal rat cardiomyocytes. These findings have important significance for further understanding the progressive and coordinated maturation of ID during the remodeling of cardiamyocytes in EHTs.Since cardiomyocytes in EHTs live in a 3D microenvironment provided by biomaterials, the development mode of the ID during the remodeling of cardiomyocytes may be affected by the used biomaterials. Both collagen and Matrigel are derived from ECM. Collagen has special amino acid sequences which can be recognized by cells [13], [22] and constitutes the skeleton structure of the substrate material. Meanwhile, a broad range of basement membrane proteins supplied by Matrigel, especially some soluble factors, can promote cell growth and benefit the remodeling of 3D tissue culture *in vitro* [13], [23]. It was reported that the presence of Matrigel is premise for cardiomyocytes to extend cell-cell contacts in collagen matrix [24]. Hence, we speculate that the 3D microenvironment provided by collagen/Matrigel, incorporating chemical signals from ECM and soluble factors, influences the behavior of the cardiomyocytes and the formation of the ID.

An important structure of cell-matrix junctions—the costamere can be observed under TEM in the EHTs at day 14 (Fig. 5c). It is rich in focal adhesion-related proteins, by which the cytoskeleton and sarcomere can be connected to the cell membranes and ECM [16]. The costamere with grid-like structure reinforces mechanical junctions and participates in force transmission and signal transduction between cell and matrix, and is closely associated with the structure and function of the ID [16], [25]. Because the costamere is a critical cytoskeletal element involved in cardiomyocyte mechanotransduction, and meanwhile the ID (e.g. gap junction) can be regulated by extracellular mechanical forces by its linkage to the cytoskeleton [26], [27]. Therefore, based on the above mentioned, the presence of the costamere in the EHTs reveals there is direct interaction between collagen/Matrigel matrix and cardiomyocytes, and it suggests that collagen/Matrigel matrix could influence cardiomyocytes and the ID by the costamere structure.

We used fluorescence intensity assay and western blotting method to quantitatively analyze the expression of the ID-related proteins. The results indicate a close correlation between the expression of functional proteins and their spatial distribution [28], [29]. In the EHTs built by collagen/Matrigel matrix at day 3, the proteins for mechanical junctions in a relatively high expression level is ready for the functional distribution. Day 7 is a critical time point of the localized distribution, when their expression significantly reduces. At day 14, further development leads to a sharp increase. For Cx43, at day 14 when obvious localized distribution appears, its expression significantly decreases. These findings imply that it may be helpful to use quantitative analysis of ID-related proteins to evaluate the quality and function of EHTs. In addition, the expression of the four proteins in 2D culture obviously increases from day 3 to day 7, but slightly decreases or remains unchanged at day 14, different from that in the EHTs. This may be due to the progressive disintegration of IDs in neonatal rat cardiomyocytes growing in 2D culture [19].

In conclusion, the mode of spatiotemporal development of the ID in collagen/Matrigel matrix is proposed: the development of the ID accompanies the remodeling of cardiomyocytes in collagen/Matrigel matrix; all the related proteins are recruited from uniform distribution to lateral aggregation along the axis of contraction, and the formation of adherens connections and desmosomes is earlier than that of gap junctions. In the EHTs, 3D microenvironment provided by collagen/Matrigel matrix plays a great role in the ordered and integrated formation of the ID.
